# Baseline Perfusion Index as a Predictor of Post-spinal Hypotension in Elective Caesarean Section: A Prospective Observational Study

**DOI:** 10.7759/cureus.109506

**Published:** 2026-05-23

**Authors:** Kazi Samsuzzaman, Prithviraj Chakraverty, Subhashis Saha, Anuradha Mitra, Madhumita Bhakta

**Affiliations:** 1 Department of Anaesthesiology, Belle Vue Clinic, Kolkata, IND; 2 Department of Anaesthesiology, KPC Medical College and Hospital, Kolkata, IND; 3 Department of Community Medicine, SLN (Saheed Laxman Nayak) Medical College and Hospital, Koraput, IND; 4 Community Medicine, MKCG (Maharaja Krishna Chandra Gajapati) Medical College and Hospital, Berhampur, IND

**Keywords:** caesarean section, hypotension, obstetric anaesthesia, perfusion index, spinal anaesthesia

## Abstract

Introduction

Spinal anaesthesia is the preferred technique for cesarean delivery, but it is frequently associated with hypotension, leading to adverse maternal and fetal outcomes. Early prediction remains a clinical challenge. Perfusion index (PI), a non-invasive marker of peripheral perfusion, has emerged as a potential predictor.

Methodology

This prospective observational study included 100 parturients undergoing elective lower segment caesarean section under spinal anaesthesia. Baseline PI was recorded preoperatively, and patients were categorized into two groups: PI ≤3.5 and PI >3.5. Hypotension was defined as a mean arterial pressure of <65 mmHg. Hemodynamic parameters, vasopressor requirement, and fluid administration were recorded. Regression and ROC curve analysis were performed.

Results

The incidence of hypotension was significantly higher in the PI >3.5 group (84%) as compared to the PI ≤3.5 group (12%) (p < 0.001). Baseline PI was a strong predictor of hypotension (OR 4.69, p < 0.001). The model demonstrated good discriminative ability (AUC = 0.895), with sensitivity of 75% and specificity of 88.5%. Higher PI was also associated with increased vasopressor and fluid requirements.

Conclusion

Baseline PI is a reliable, non-invasive predictor of post-spinal hypotension in parturients. Its incorporation into routine preoperative assessment may enable early risk stratification and targeted haemodynamic management.

## Introduction

Spinal anesthesia is the preferred anesthetic technique for lower-segment cesarean section (LSCS) because of its rapid onset, reliable sensory and motor blockade, and minimal neonatal drug exposure. However, maternal hypotension remains the most common and clinically significant complication associated with spinal anesthesia. The incidence of hypotension in parturients undergoing spinal anesthesia ranges from 60% to 80%, which is substantially higher than that seen in the general surgical population [1,2]. The main mechanism of spinal anesthesia-induced hypotension is sympathetic blockade, resulting in peripheral vasodilation, reduced systemic vascular resistance, venous pooling, and decreased venous return, ultimately leading to reduced cardiac output and hypotension [3]. Physiological changes during pregnancy, including reduced vascular tone and aortocaval compression by the gravid uterus, further predispose parturients to hemodynamic instability [4]. Pregnant women are also more sensitive to local anesthetics and less responsive to vasopressors, increasing their vulnerability to severe hypotension [5]. Maternal hypotension during cesarean section can lead to nausea, vomiting, dizziness, and impaired uteroplacental perfusion, resulting in fetal hypoxia, acidosis, and adverse neonatal outcomes [6-8]. Even short episodes of hypotension may produce fetal bradycardia and acid-base disturbances, emphasizing the importance of early prediction and management [9].

Despite advances in obstetric anesthesia, predicting hypotension following spinal anesthesia remains difficult. Conventional monitoring methods, such as intermittent non-invasive blood pressure (NIBP) monitoring, may fail to identify rapid hemodynamic fluctuations and provide limited information about peripheral perfusion [10]. Several predictive methods, including baseline heart rate, heart rate variability, and ultrasound-based indices, have been investigated, but these techniques are often inconsistent, operator-dependent, or impractical for routine use [11,12].

Perfusion index (PI), a non-invasive parameter derived from pulse oximetry, has recently emerged as a potential predictor of spinal anaesthesia-induced hypotension. PI represents the ratio of pulsatile to non-pulsatile blood flow in peripheral tissues and reflects peripheral perfusion and vascular tone [13]. Higher PI values indicate lower peripheral vascular resistance and greater vasodilation, predisposing patients to hypotension following sympathetic blockade. Previous studies have demonstrated the usefulness of baseline PI in predicting post-spinal hypotension. Toyama et al. reported that a baseline PI greater than 3.5 was associated with a significantly higher incidence of hypotension, with good sensitivity and specificity [14]. Similarly, Duggappa et al. found that parturients with PI >3.5 had a markedly increased incidence of hypotension and greater vasopressor requirement following spinal anaesthesia [15]. Subsequent studies have supported these findings, highlighting PI as a simple, rapid, and non-invasive bedside predictor [16-18]. A recent meta-analysis by Hung et al. also demonstrated moderate-to-good diagnostic accuracy of PI, with pooled sensitivity and specificity of 0.81 and 0.75, respectively [19]. However, important limitations persist in the existing literature. Considerable variability exists in reported PI cut-off values, ranging from 1.75 to 3.9, likely due to differences in patient populations and study methodologies [17,20]. Many studies have relatively small sample sizes and lack standardised definitions of hypotension. Furthermore, limited evidence is available from the Indian population, where demographic and physiological differences may influence the predictive value of PI. Therefore, the present study was undertaken to evaluate baseline PI as a predictor of hypotension following spinal anaesthesia in parturients undergoing elective cesarean section. By correlating baseline PI with hemodynamic outcomes and assessing its predictive accuracy, the study aims to facilitate early risk stratification and improve perioperative hemodynamic management in obstetric anaesthesia.

The primary objective of this study was to evaluate baseline PI as a predictor of hypotension following spinal anaesthesia in parturients undergoing elective LSCS. Secondary objectives included assessing the association between PI and vasopressor requirement, fluid administration, and frequency of hypotensive episodes. Through this approach, the study seeks to determine whether PI can be incorporated into routine clinical practice as a simple, non-invasive tool for optimising maternal and fetal outcomes.

We hypothesised that a higher baseline PI (>3.5) would be associated with an increased incidence of hypotension following spinal anaesthesia in parturients undergoing elective cesarean section.

## Materials and methods

This study was conducted as a prospective observational study to evaluate the predictive role of baseline PI in determining the occurrence of hypotension following spinal anesthesia in parturients undergoing elective lower-segment cesarean section (LSCS). The study was carried out in the Department of Anesthesiology at a tertiary care teaching hospital, where elective cesarean sections are routinely performed under spinal anesthesia with standardized perioperative monitoring and management protocols. The study was conducted over a defined period sufficient to recruit the required sample size, including phases of patient enrolment, intraoperative monitoring, data collection, and analysis. The study population comprised parturients scheduled for elective LSCS under spinal anesthesia. Only patients meeting predefined eligibility criteria were included to ensure homogeneity and minimize confounding variables that could affect hemodynamic responses.

Sample size was calculated based on the anticipated incidence of post-spinal hypotension in parturients undergoing cesarean section, estimated at 60% from previous literature. Using a 95% confidence level, an allowable error of 10%, and accounting for possible exclusions and dropouts, the minimum sample size was estimated at 92. A total of 100 participants were included to ensure adequate statistical power. A consecutive sampling technique was employed, wherein a total of 118 patients presenting during the study period were assessed for eligibility. Twelve patients did not meet the inclusion criteria, and six declined participation. Finally, 100 eligible parturients were enrolled and analyzed. Pregnant women aged 20-35 years, belonging to American Society of Anesthesiologists (ASA) physical status II [21], with singleton term pregnancies (36-41 weeks of gestation), and scheduled for elective LSCS under spinal anesthesia were included in the study. Parturients with hypertensive disorders of pregnancy (including preeclampsia and eclampsia), placenta previa, cardiovascular or cerebrovascular diseases, gestational diabetes mellitus, or any contraindication to spinal anesthesia were excluded. Additionally, patients with body mass index (BMI) greater than 40 kg/m², gestational age outside 36-41 weeks, or those requiring emergency cesarean section were not included.

After obtaining informed consent, baseline hemodynamic parameters, including heart rate (HR), non-invasive blood pressure (NIBP), oxygen saturation (SpO₂), and PI, were recorded in the supine position with left uterine displacement. An intravenous line was secured, and all participants received a standardized preload of Ringer’s lactate 10 mL/kg prior to spinal anesthesia. Spinal anesthesia was administered in the left lateral position at the L3-L4 or L2-L3 interspace using a 25-gauge Quincke needle, and 10 mg of 0.5% hyperbaric bupivacaine was injected intrathecally. Following the block, patients were positioned supine with a 15° left lateral tilt. Sensory block level was assessed after 5 minutes, and patients not achieving a T6 level were excluded. Based on baseline PI values, patients were categorized into two groups: Group 1 (PI ≤3.5) and Group 2 (PI >3.5). The anesthesiologist administering spinal anesthesia was blinded to PI values to avoid bias. PI was measured using a standard pulse oximeter probe placed on the index finger. Hemodynamic parameters were monitored using multiparameter monitors, including automated NIBP devices. Hemodynamic variables (heart rate (HR), systolic blood pressure (SBP), diastolic blood pressure (DBP), mean arterial pressure (MAP), peripheral oxygen saturation (SpO₂), and PI) were recorded at baseline and subsequently at 5-minute intervals for the first 20 minutes following spinal anesthesia. The occurrence of hypotension, the number of hypotensive episodes, the total dose of vasopressor (ephedrine), and fluid requirement were documented. Neonatal outcome was assessed using Apgar scores at 1 and 5 minutes. The primary outcome was the incidence of hypotension following spinal anesthesia. Hypotension was defined as MAP <65 mmHg, consistent with previous studies demonstrating impaired organ perfusion below this threshold. MAP-based definition was preferred, as it reflects systemic perfusion more reliably than isolated systolic blood pressure measurements. Secondary outcomes included the association of baseline PI with vasopressor requirement, total fluid administered, and frequency of hypotensive episodes.

All data were entered into Microsoft Excel (Microsoft Corporation, Redmond, WA, US) and analyzed using JAMOVI version 2.6.44 (www.jamovi.org) and R Studio version 4.5.3 (R Core Team, Vienna, Austria). Continuous variables were expressed as mean ± standard deviation, while categorical variables were presented as frequencies and percentages. Normality of continuous variables was assessed using the Shapiro-Wilk test prior to parametric analysis. Comparisons between groups were performed using an independent t-test and a chi-square test. Model calibration was assessed using the Hosmer-Lemeshow goodness-of-fit test. Regression analysis was used to assess the predictive value of PI, and receiver operating characteristic (ROC) curve analysis was performed to evaluate diagnostic accuracy. A p-value <0.05 was considered statistically significant. To ensure accuracy and reliability, all measurements were performed by trained personnel using calibrated equipment. Standardized protocols were followed for anesthesia administration and monitoring. Data entry was cross-verified to minimize errors.

The study was conducted following approval from the Institutional Ethics Committee No. KPCMCH/IEC/2023/98. Written informed consent was obtained from all participants prior to enrolment. Confidentiality of patient data was maintained throughout the study, and participation was entirely voluntary and did not affect standard clinical care.

## Results

Baseline characteristics of the study population

A total of 100 parturients were included in the study and were equally divided into two groups based on baseline PI: Group 1 (PI ≤3.5, n=50) and Group 2 (PI >3.5, n=50).

The mean age of participants in Group 1 was 27.6 ± 4.02 years, while in Group 2, it was 27.5 ± 3.91 years. The difference was not statistically significant (p = 0.94), indicating comparable age distribution between the groups. Similarly, the mean body weight was 57.1 ± 4.89 kg in Group 1 and 57.4 ± 5.15 kg in Group 2, with no statistically significant difference (p = 0.766). These findings confirm that both groups were well-matched with respect to baseline demographic characteristics (Table 1). The baseline demographic variables, including age and weight, were comparable between the two groups (p > 0.05), indicating homogeneity of the study population. As expected, the baseline PI was significantly higher in Group 2 (p < 0.001), confirming appropriate group stratification. No significant difference in Apgar scores at 1 and 5 minutes was observed between groups.

**Table 1:** Baseline characteristics of the study population (N=100) *Statistically significant at p <0.05.

Variable	Group 1 (PI ≤3.5) (n=50)	Group 2 (PI >3.5) (n=50)	p-value
Age (years)	27.6 ± 4.02	27.5 ± 3.91	0.94
Age Range (years)	20 – 34	20 – 34	—
Weight (kg)	57.1 ± 4.89	57.4 ± 5.15	0.766
Weight Range (kg)	52 – 69	52 – 69	—
Baseline PI	2.8 ± 0.42	4.95 ± 1.18	<0.001*

Baseline PI distribution

**Figure 1:**
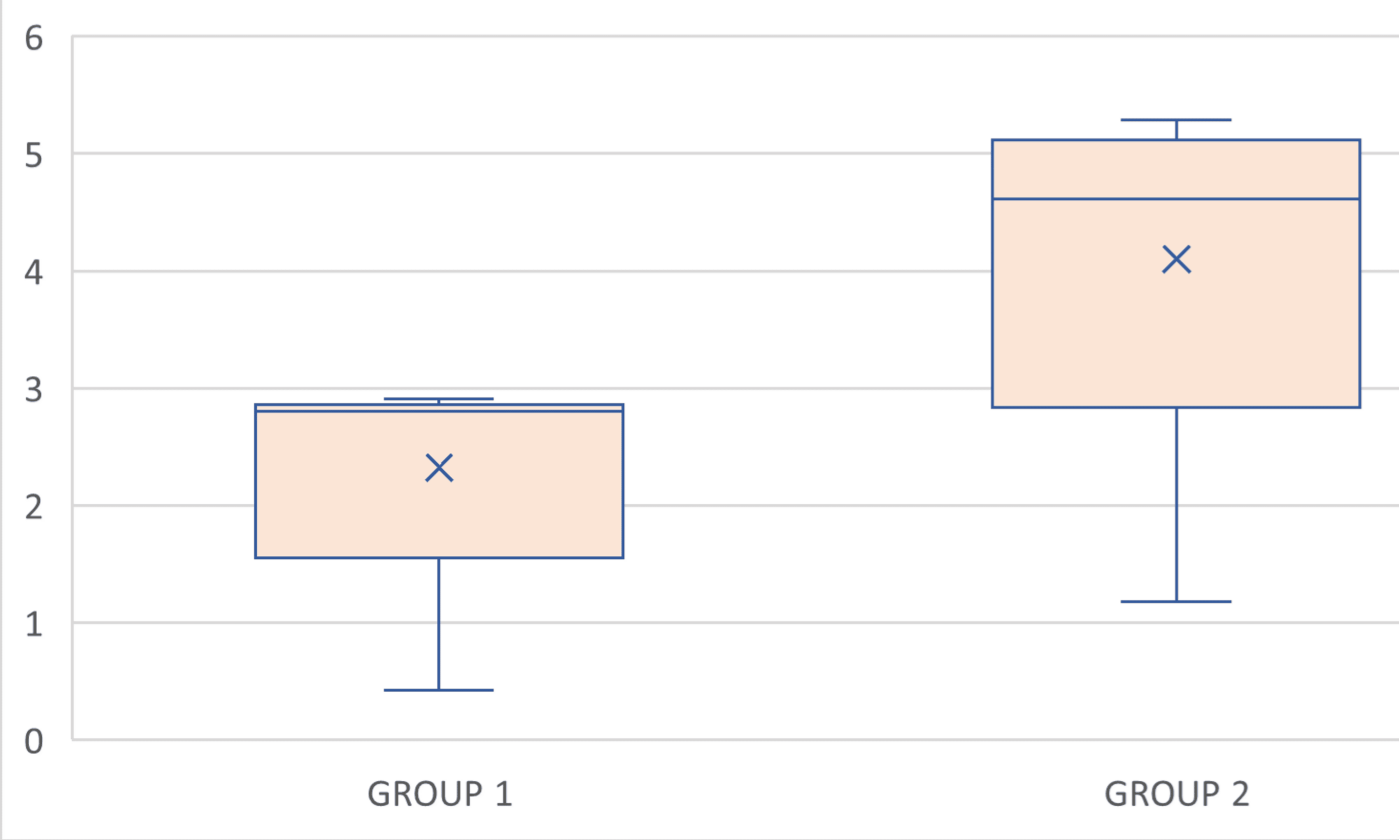
Baseline perfusion index

The baseline PI differed significantly between the two groups by design. Group 1 had a mean PI of 2.8 ± 0.42, whereas Group 2 had a significantly higher mean PI of 4.95 ± 1.18. The distribution of PI values confirmed clear stratification between the groups, with minimal overlap (Figure 1).

The overall incidence of hypotension was 48% (48/100). However, a marked difference was observed between the groups. In Group 1 (PI ≤3.5), only 6 patients (12%) developed hypotension, whereas in Group 2 (PI >3.5), 42 patients (84%) experienced hypotensive episodes. This difference was highly statistically significant (χ² = 51.9, df=2, p < 0.001), demonstrating a strong association between higher baseline PI and increased risk of hypotension following spinal anaesthesia. The number of hypotensive episodes was significantly greater in Group 2 as compared to Group 1. In Group 1, 88% of patients experienced no hypotension, and only a small proportion had 1 or more episodes. In contrast, Group 2 demonstrated a higher frequency of multiple hypotensive episodes, with several patients experiencing two or more events. The difference in distribution of hypotensive episodes between the two groups was statistically significant (χ² = 52.9, df=4, p < 0.001) (Table 2). The requirement for vasopressor (ephedrine) was significantly higher in Group 2 as compared to Group 1. The mean dose of ephedrine administered in Group 1 was 2.78 ± 1.95 mg, whereas in Group 2, it was 18.1 ± 3.89 mg. This difference was statistically significant (p < 0.01), indicating that patients with higher baseline PI required greater pharmacological support to maintain haemodynamic stability. Patients in Group 2 required a significantly higher intravenous fluid administration compared to those in Group 1. The mean fluid requirement was 992 ± 56.9 mL in Group 1 and 1064 ± 43.9 mL in Group 2, with a statistically significant difference (p < 0.01). This finding further supports the association between higher baseline PI and increased haemodynamic instability.

**Table 2:** Comparison of hemodynamic outcomes between groups (N=100) *Statistically significant at p <0.05.

Parameter	Group 1 (PI ≤3.5) (n=50)	Group 2 (PI >3.5) (n=50)	p-value
Incidence of hypotension (n (%))	6 (12%)	42 (84%)	<0.001* —
No hypotension (n (%))	44 (88%)	8 (16%)	
Number of hypotensive episodes (n (%))			<0.001*
0 episodes	44 (88%)	8 (16%)	
1 episode	4 (8%)	18 (36%)	
2 episodes	1 (2%)	16 (32%)	
3 episodes	1 (2%)	4 (8%)	
4 episodes	0 (0%)	4 (8%)	
Vasopressor requirement (Ephedrine, mg)	2.78 ± 1.95	18.1 ± 3.89	<0.01*
Fluid requirement (mL)	992 ± 56.9	1064 ± 43.9	<0.01*

Predictive value of baseline PI

**Figure 2:**
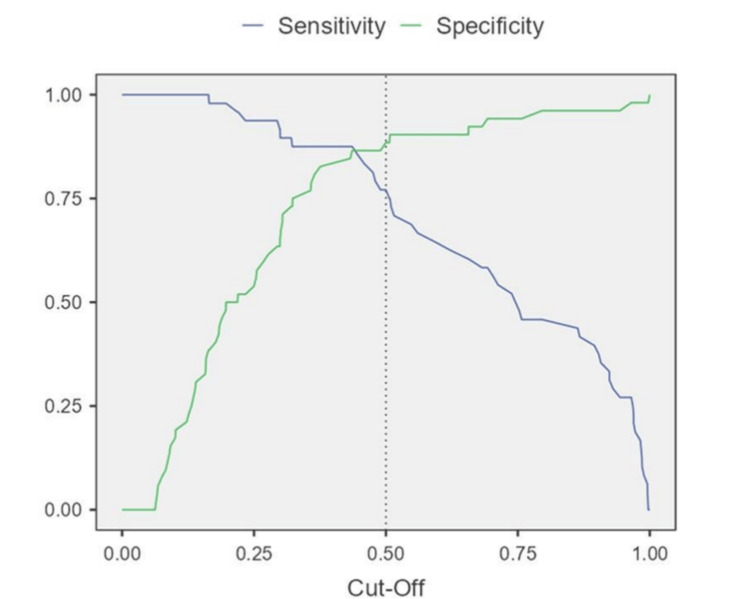
Cut-off plot for predictive measures

**Figure 3:**
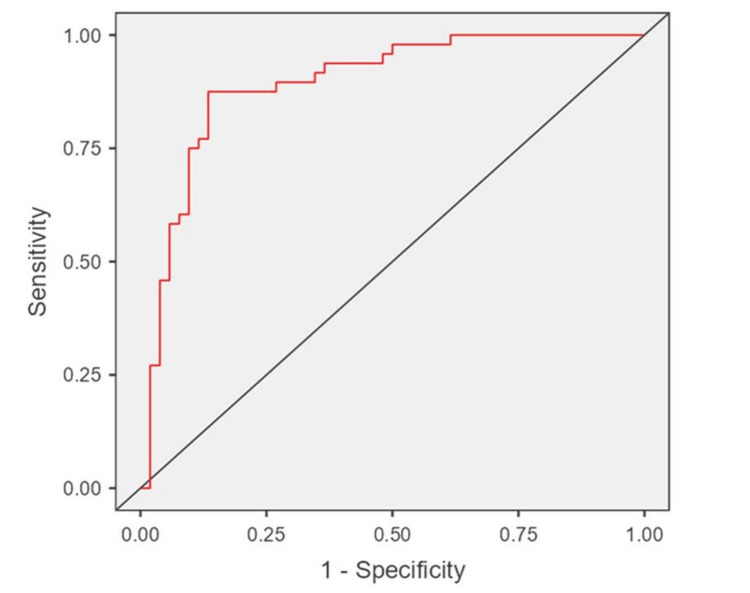
ROC curve showing area under the curve ROC: receiver operating characteristic

The model showed a good fit with a McFadden’s R² of 0.347. The classification accuracy was 82%, with a sensitivity of 75% and a specificity of 88.5%. Receiver operating characteristic (ROC) curve analysis demonstrated excellent discriminative ability of baseline PI, with an area under the curve (AUC) of 0.895 (95% CI: 0.82-0.96) (Table 3, Figures 2, 3). Although internal validation using bootstrapping or split-sample validation was not performed, the predictive model demonstrated good discrimination.

**Table 3:** Model predictor for hypotension episode *Statistically significant at p <0.05.

Parameter	Value
Model Fit Measures	
Deviance	90.4
AIC	94.4
McFadden’s R²	0.347
Logistic Regression Coefficients for Hypotension	
Predictor	Baseline PI
Estimate (β)	1.55
Standard Error (SE)	0.326
95% Confidence Interval	0.908 – 2.19
Z value	4.74
p-value	<0.001*
Odds Ratio (OR)	4.69
Classification Performance	
Accuracy	82%
Sensitivity	75%
Specificity	88.5%
Area Under Curve (AUC)	0.895

A multivariable multinomial logistic regression model was constructed to evaluate the independent association of baseline PI, age, body weight, ephedrine dose, and fluid requirement with the frequency of hypotensive episodes following spinal anaesthesia. Patients with no hypotensive episodes were considered the reference category. The overall model demonstrated good statistical significance (χ² = 70.2, df = 20, p <0.001), indicating that the included predictors significantly improved model fit. The model showed moderate explanatory power, with McFadden’s pseudo-R² of 0.28, Cox and Snell R² of 0.131, and Nagelkerke R² of 0.332. The deviance, Akaike Information Criterion (AIC), and Bayesian Information Criterion (BIC) values were 180, 228, and 291, respectively, suggesting acceptable model adequacy (Table 4) [22,23].

**Table 4:** Model fit of measures for multivariable multinomial logistic regression analysis for hypotension episodes Models estimated using a sample size of N=100. AIC: Akaike Information Criterion; BIC: Bayesian Information Criterion

Model Fit Measures								
						Overall Model Test		
Deviance	AIC	BIC	R²McF	R²CS	R²N	χ²	df	p
180	228	291	0.28	0.131	0.332	70.2	20	< .001

For patients experiencing one hypotensive episode, baseline PI showed a positive association with hypotension frequency (OR = 2.17), although the association approached but did not achieve statistical significance (p = 0.057). Age, body weight, ephedrine dose, and fluid requirement were not independently associated with this category. Among patients with two hypotensive episodes, baseline PI emerged as a statistically significant predictor (β = 0.86, OR = 2.37, 95% CI: 1.05-5.38, p = 0.039). The dose of ephedrine was also significantly associated with this category (OR = 1.16, p = 0.024), indicating greater vasopressor requirement in patients with recurrent hypotension. For patients experiencing three hypotensive episodes, baseline PI demonstrated a strong and statistically significant association (β = 1.92, OR = 6.79, 95% CI: 2.03-22.75, p = 0.002). This finding indicates that higher baseline PI markedly increased the likelihood of recurrent hypotensive episodes. Other covariates were not independently significant in this category. In patients with four hypotensive episodes, baseline PI demonstrated a positive but non-significant association (OR = 2.91, p = 0.091). However, the ephedrine requirement remained significantly associated with recurrent hypotension (OR = 1.29, p = 0.019). Fluid requirement demonstrated a significant inverse association (OR = 0.97, p <0.001), suggesting that greater fluid administration may have partially attenuated severe, recurrent hypotensive episodes (Table 5).

**Table 5:** Multivariable multinomial logistic regression analysis for hypotension episodes

Model Coefficients - Episodes of Hypotension								
			95% Confidence Interval					
Episodes of Hypotension	Predictor	Estimate	Lower	Upper	SE	Z	p	Odds ratio
1 - 0	Intercept	-9.14849	-9.15595	-9.14102	0.00381	-2400.73	< .001	1.06E-04
	PI baseline	0.77327	-0.0215	1.56805	0.40551	1.907	0.057	2.167
	Age (YRS)	-0.01151	-0.16826	0.14524	0.07998	-0.144	0.886	0.989
	Weight (KG)	0.04136	-0.0619	0.14463	0.05269	0.785	0.432	1.042
	Dose of ephedrine	0.09193	-0.01468	0.19855	0.0544	1.69	0.091	1.096
	Fluid requirement in mL	0.00239	-0.00401	0.00879	0.00327	0.731	0.465	1.002
2 - 0	Intercept	-8.27807	-8.2861	-8.27005	0.0041	-2021.33	< .001	2.54E-04
	PI baseline	0.86392	0.04444	1.6834	0.41811	2.066	0.039	2.372
	Age (yrs)	0.05179	-0.1281	0.23168	0.09178	0.564	0.573	1.053
	Weight (kg)	-0.05428	-0.19565	0.08708	0.07213	-0.753	0.452	0.947
	Dose of ephedrine	0.14682	0.01896	0.27467	0.06523	2.251	0.024	1.158
	Fluid requirement in mL	0.00356	-0.00435	0.01147	0.00404	0.881	0.378	1.004
3 - 0	Intercept	-14.8321	-14.8523	-14.812	0.01028	-1442.31	< .001	3.62E-07
	PI baseline	1.9158	0.70698	3.12461	0.61675	3.106	0.002	6.792
	Age (yrs)	0.08854	-0.19521	0.37228	0.14477	0.612	0.541	1.093
	Weight (kg)	0.13446	-0.04521	0.31413	0.09167	1.467	0.142	1.144
	Dose of ephedrine	-0.0922	-0.30688	0.12248	0.10953	-0.842	0.4	0.912
	Fluid requirement in mL	-0.00448	-0.01785	0.00889	0.00682	-0.656	0.512	0.996
4 - 0	Intercept	21.80608	21.80414	21.80801	9.88E-04	22078.27	< .001	2.95E+09
	PI baseline	1.06773	-0.17001	2.30547	0.63151	1.691	0.091	2.909
	Age (yrs)	-0.19133	-0.51539	0.13272	0.16534	-1.157	0.247	0.826
	Weight (kg)	0.08384	-0.1161	0.28377	0.10201	0.822	0.411	1.087
	Dose of ephedrine	0.25055	0.04127	0.45982	0.10677	2.346	0.019	1.285
	Fluid requirement in mL	-0.03092	-0.04351	-0.01834	0.00642	-4.816	< .001	0.97

The multivariable multinomial regression analysis demonstrated that baseline PI remained an important independent predictor of increasing frequency and severity of hypotensive episodes following spinal anaesthesia. The progressively increasing odds ratios across higher hypotension categories further support the clinical utility of baseline PI as a predictor of hemodynamic instability in parturients undergoing elective caesarean section.

## Discussion

The present prospective observational study evaluated baseline PI as a predictor of hypotension following spinal anaesthesia in parturients undergoing elective lower segment caesarean section. The findings demonstrate that baseline PI is a clinically relevant predictor of post-spinal hypotension with important implications for perioperative haemodynamic management. The baseline demographic characteristics, including age and body weight, were comparable between the two groups, minimising the confounding effects of these variables on haemodynamic outcomes. Previous studies have shown that maternal age and body weight can influence cardiovascular responses to spinal anaesthesia through variations in autonomic tone and circulating blood volume [3,4]. However, the comparable distribution of these variables in the present study strengthens the validity of the observed association between PI and hypotension. Similar findings have been reported in earlier studies evaluating PI as a predictive marker [15,20].

The incidence of hypotension was significantly higher in patients with baseline PI >3.5 (84%) compared to those with PI ≤3.5 (12%). This finding is physiologically plausible, as spinal anaesthesia causes sympathetic blockade leading to vasodilation, reduced systemic vascular resistance, venous pooling, and decreased cardiac output [3]. Pregnancy-related physiological changes, such as reduced vascular tone and aortocaval compression, further increase susceptibility to hypotension [4,5]. Since PI reflects peripheral vascular tone, higher baseline PI values indicate pre-existing vasodilation, predisposing patients to greater haemodynamic instability following spinal anaesthesia. The findings of the present study are consistent with those of Toyama et al., who demonstrated that a baseline PI greater than 3.5 was associated with significantly increased incidence of hypotension [14]. Similarly, Duggappa et al. reported higher rates of hypotension and vasopressor requirement among parturients with elevated baseline PI values [15]. Other studies have also supported the utility of PI as a simple and non-invasive predictor of spinal anaesthesia-induced hypotension [16-18]. In addition to incidence, the present study demonstrated a higher frequency of hypotensive episodes in patients with higher PI values, suggesting that PI predicts not only the occurrence but also the severity of hypotension. Similar observations have been reported previously, where elevated PI correlated with greater haemodynamic instability [15,17]. Patients with higher baseline PI also required significantly greater vasopressor and fluid administration. Increased ephedrine requirement in the high PI group indicates more severe hypotension requiring pharmacological intervention. These findings are consistent with earlier reports demonstrating increased vasopressor requirement in patients with elevated PI values [15,18]. Ngan Kee et al. emphasised the importance of timely vasopressor therapy in maintaining maternal blood pressure and uteroplacental perfusion during spinal anaesthesia [6]. Similarly, higher fluid requirements observed in the present study reflect greater vasodilation and reduced preload associated with sympathetic blockade [6,9]. Previous studies have also shown that patients with elevated PI require greater fluid resuscitation to maintain haemodynamic stability [17,20]. Logistic regression analysis further strengthened the predictive role of baseline PI. The odds ratio of 4.69 indicated that each unit increase in PI significantly increased the likelihood of developing hypotension. The predictive model demonstrated excellent discriminative ability, with an area under the ROC curve (AUC) of 0.895. These findings are comparable to previous studies reporting AUC values ranging from 0.84 to 0.91 for PI in predicting post-spinal hypotension [19,20]. The high specificity observed in the present study suggests that PI may be useful for identifying patients at low risk of hypotension and facilitating perioperative risk stratification.

PI offers several clinical advantages. It is a simple, non-invasive, and readily available parameter that can be measured using standard pulse oximetry without additional expertise or equipment. Unlike other predictors, such as heart rate variability or ultrasound-based indices, PI is easy to integrate into routine obstetric anaesthesia practice [11,12]. Early identification of high-risk patients may facilitate targeted interventions, including optimised fluid management, prophylactic vasopressor therapy, and closer haemodynamic monitoring.

The present study has certain limitations. It was conducted at a single centre with a relatively modest sample size, which may limit generalizability. In addition, invasive haemodynamic monitoring and long-term neonatal outcomes were not assessed.

## Conclusions

Baseline perfusion index (PI) was found to be a promising and clinically useful predictor of hypotension following spinal anaesthesia in parturients undergoing elective caesarean section. Patients with higher baseline PI values (>3.5) demonstrated a significantly increased incidence, frequency, and severity of hypotension, along with greater vasopressor and fluid requirements. The strong association observed, supported by logistic regression and ROC analysis, highlights the excellent predictive accuracy of PI in identifying high-risk patients preoperatively. Given its ease of measurement using standard pulse oximetry, PI can be readily incorporated into routine clinical practice without additional cost or expertise. Early identification of patients at risk allows for targeted prophylactic strategies, including optimised fluid management and timely vasopressor use, thereby improving maternal haemodynamic stability and potentially enhancing fetal outcomes. Further multicentric studies with larger populations are recommended to validate these findings and establish standardised PI cut-off values.
